# Inertial measurement and heart-rate sensor-based dataset for geriatric fall detection using custom built wrist-worn device

**DOI:** 10.1016/j.dib.2023.109812

**Published:** 2023-11-18

**Authors:** Purab Nandi, K. R Anupama, Himanish Agarwal, Kishan Patel, Vedant Bang, Manan Bharat, Madhen Vyas Guru

**Affiliations:** BITS Pilani, K K Birla, Goa Campus, Goa 403726, India

**Keywords:** Geriatric, Fall detection, Wearable, System on Chip, Dataset

## Abstract

This paper describes a dataset acquired from 41 volunteers performing 16 Activities of daily livings (ADLs) and 8 Falls repeated 5 times. This data was collected using a custom wrist-worn end device. The dataset has data collected from Inertial measurement unit (IMU) and heart-rate sensors. The end device is built using Qualcomm Snapdragon 820c System on Chip (SoC) interfaced to the sensors via Interconnect Integrated Circuit (I2C) protocol. The data was sampled for every activity at a rate of 20 Hz for the motion sensors and at a rate of 1 Hz for the heart-rate sensor. The motion sensor comprised of a triaxial accelerometer, triaxial gyroscope, triaxial magnetometer and a linear accelerometer. The heart-rate sensor was medical grade and all sensors were calibrated for the wrist -worn position. The dataset is available on this website https://shamanx86.github.io/fall_detection_data/ and https://doi.org/10.5281/zenodo.10013090.

Specifications Table

**Table:** 

Subject	Computer Science in Healthcare
Specific subject area	Geriatric Fall detection
Data format	Raw data of triaxial accelerometer, triaxial gyroscope, triaxial magnetometer, linear accelerometer and Heart-rate sensor with time stamp.
Type of data	Table, each table having six columns, time-stamp, x axis data, y-axis data and z axis data, number of axis and type of sensor(label) except in case of heart-rate where there will be only three columns, time-stamp, beats per minute and label (hrt for heart-rate)
Data collection	Data was collected using a custom built-wrist worn end worn on the left-wrist. Qualcomm Snapdragon 820c. We used MAX30102 Heart rate and SP02 sensor, MPU6500, which gives 3-axis acceleration, 3-axis linear acceleration and 3-axis gyroscope data and GY273 Magnetometer chip for data collection. All the sensors are interfaced to the SoC via the I2C interface using a Mezzanine board.
Data source location	Birla Institute of Technology and Science, Pilani – K.K Birla Goa Campus, Goa, India.
Data accessibility	• Direct URL to data: https://shamanx86.github.io/fall_detection_data/ and https://doi.org/10.5281/zenodo.10013090 • Instructions for accessing these data: given in article below • All the codes are available at: https://zenodo.org/badge/latestdoi/517690954

## 1 Value of the Data


 

• This is the only dataset available that uses only wrist worn data and has a total of 4920 instances.
• Though there are other wearable based datasets available, the sensors are worn either as a combination of the thigh, waist, torso, ankle or all of the above.
• We have thoroughly analysed the performance on various ML algorithms and obtained accuracies and precision in detecting Falls as high as 97%.
• Highly scalable as the device used to collect the data is a low-cost device that can be easily worn by any geriatric unlike image or acoustic based dataset.

## 2 Data Description

**Fig. 1:**
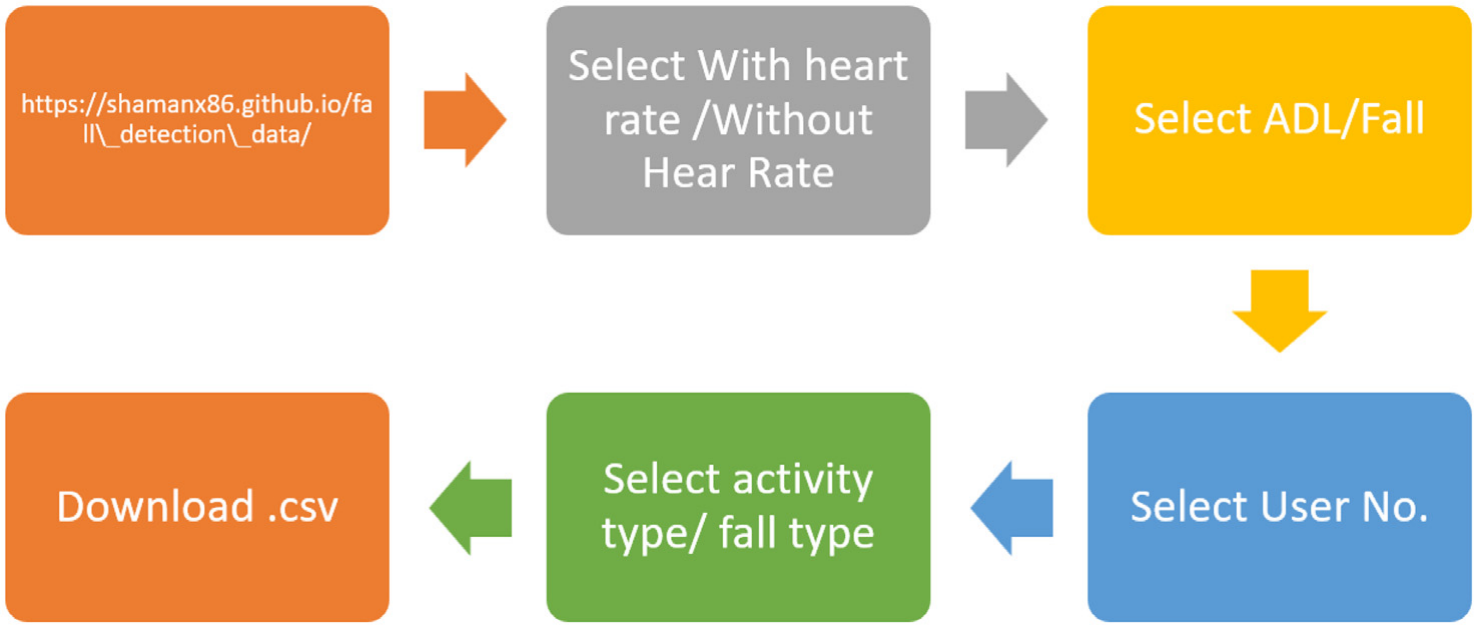
Steps to access the individual data per activity from the dataset.

The data-set and the code is available in [[1], [2]]. The steps to download the data is shown in Fig. 1.

**Fig. 2:**
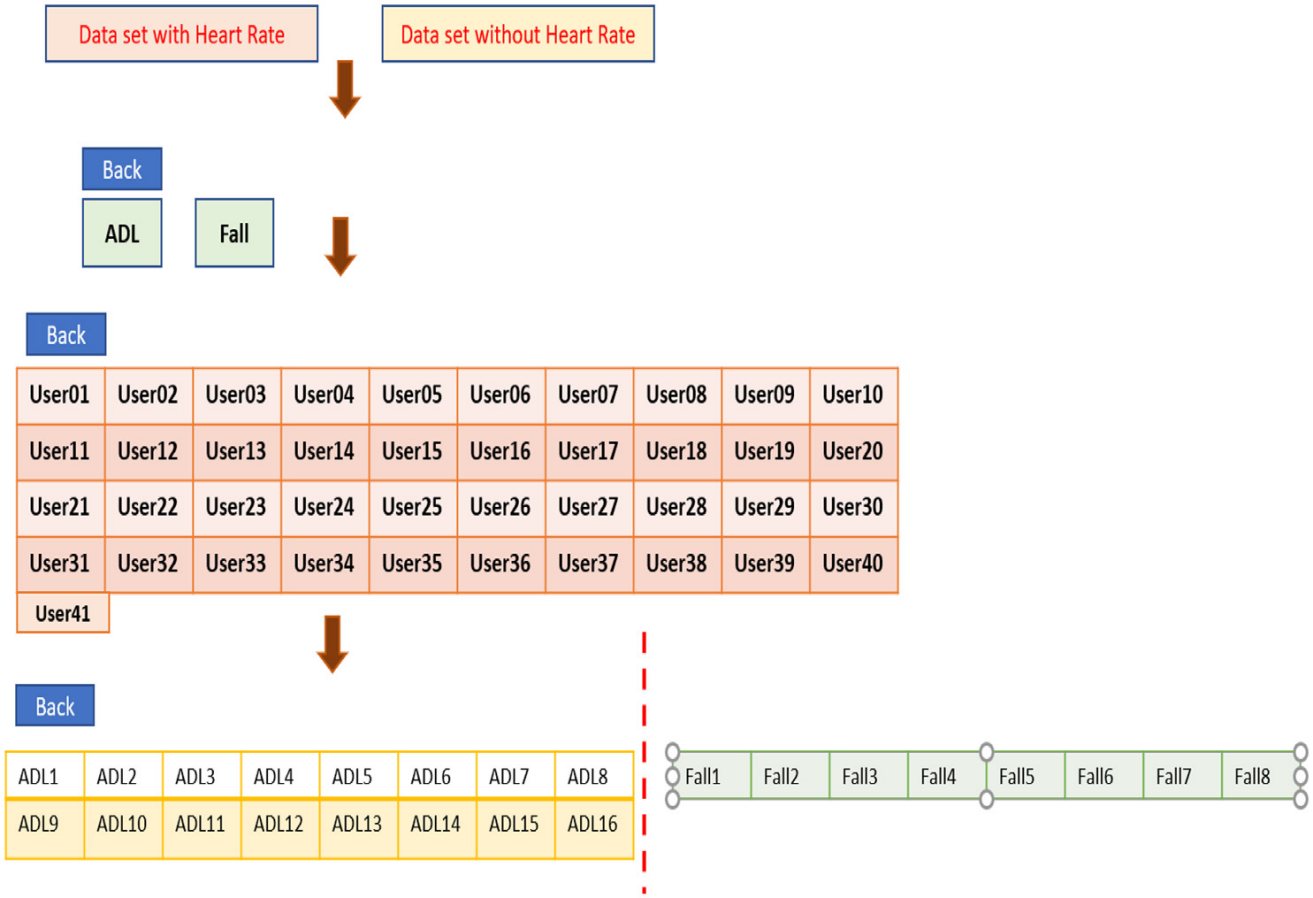
Map of the website.

The actual map to download the .csv from the website is illustrated below in Fig. 2.

Also the entire dataset in a zip file is available at: https://doi.org/10.5281/zenodo.10013090



**Format of the csv file**

**Fig. 3:**
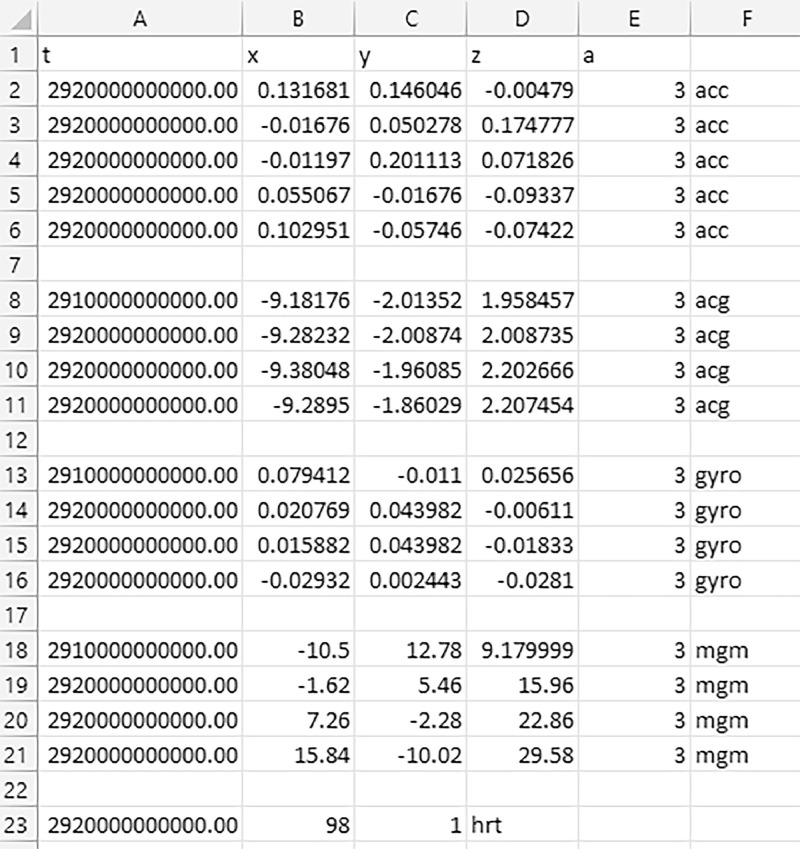
Sample dataset.

Fig. 3 gives the snapshot of the .csv file.

Column A gives the time in which the data was recorded. In case of the triaxial accelerometer, gyroscope, magnetometer and linear acceleration. The next three column i.e. B, C and D give x, y and z axis values and column E gives the number of axis and column F gives the name of the sensor in short. In case of heart-rate only four columns are used, column A is the time stamp, column B is the number of heart-beats per minute, column C indicates a single value and column D gives the sensor name in short which works as a label.

The expansion is as follows

“acc” – 3 axis accelerometer gives acceleration along x,y and z plane.

“acg” – 3 axis linear accelerometer give linear along x,y and z plane

“gyro” – 3 axis gyroscope gives the rotational moment along x,y and z plane

“mgm” – 3 axis magnetometer gives the position with respect to the earth's gravitational force along x,y and z plane

“hrt” – single value of heart-rate in beats per minute

## 3 Experimental Design, Materials and Methods

In this data article, the data was collected from a total of 41 volunteers performing 16 ADLs and 8 Falls listed in Table 1. Every activity was repeated for 5 trials.

**Table 1:** Activity list and details.

Sr No	ADL Activates	No. of Trial	Duration	Sr No	Fall Activities	No. of Trials	Duration
1	Walking Slowly	5	2 min	1	Forward Fall landing on Knee	5	40 s
2	Walking Quickly	5	2 min	2	Right Fall	5	40 s
3	Jogging	5	2 min	3	Left Fall	5	40 s
4	Jumping	5	30 s	4	Forward Fall	5	40 s
5	Climbing up slowly	5	2 min	5	Seated on Bed and falling on ground	5	40 s
6	Climbing down slowly	5	2 min	6	Forward Fall body weight on hand	5	40 s
7	Climbing up normally	5	2 min	7	Backward fall from seated position	5	40 s
8	Climbing down normally	5	2 min	8	Grabbing while falling	5	40 s
9	Slowly sitting on chair	5	30 s				
10	Rapidly sitting on chair	5	30 s				
11	Nearly sitting on chair and getting up	5	30 s				
12	Swinging Hands	5	2 min				
13	Lying on Bed	5	2 min				
14	Lying on back and getting up slowly	5	30 s				
15	Lying on back and getting up normally	5	30 s				
16	Transition from sideways to one's back while lying	5	30 s				

**Fig. 4:**
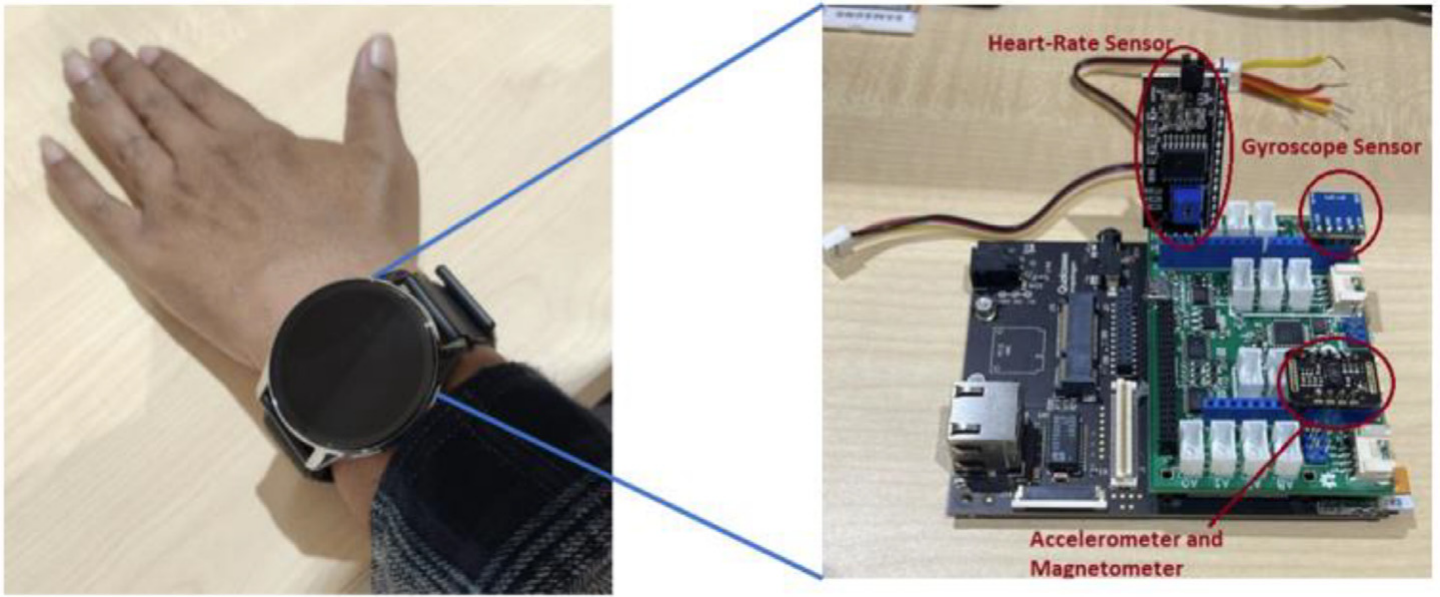
Device used for collecting data and its internal circuit.

The collection of data from various sensors was done using a python code and was perfectly timed using the inbuilt timer of the 820c. Each of the activities produced two Comma separated Variable (.csv) files, one had data from heart-rate sensor and the another which did not have the data from the heart-rate sensor. All Falls were done in the safety of a well-padded anechoic chamber. The volunteer was asked to wear the wrist-worn device on his/her left wrist while performing all the activities. Before starting each activity, the base heart-rate of the volunteer was recorded and it was ensured that they were at their base heart-rate when they started the activity. The placement of the sensor is shown in Fig. 3 below along with the internal circuitry of the wrist worn device. This system is built around a powerful System on Chip (SoC) that is Qualcomm Snapdragon 820c [3]. The 820c chip has been developed specifically for wearable and IoT applications. We used MAX30102 [4] Heart rate and SP02 sensor, MPU6500 [5], which gives 3-axis acceleration, 3-axis linear acceleration and 3-axis gyroscope data and GY273 [6] Magnetometer chip for data collection. All the sensors are interfaced to the SoC via the I2C interface using a Mezzanine board. The 820c chip also has inbuilt 802.11 transceiver to send the data wirelessly to the cloud. Also, the SD card slot can be used to store data for long durations (a week or more). The Fig. 4 gives the experimental setup of the wearable device.

**Fig. 5:**
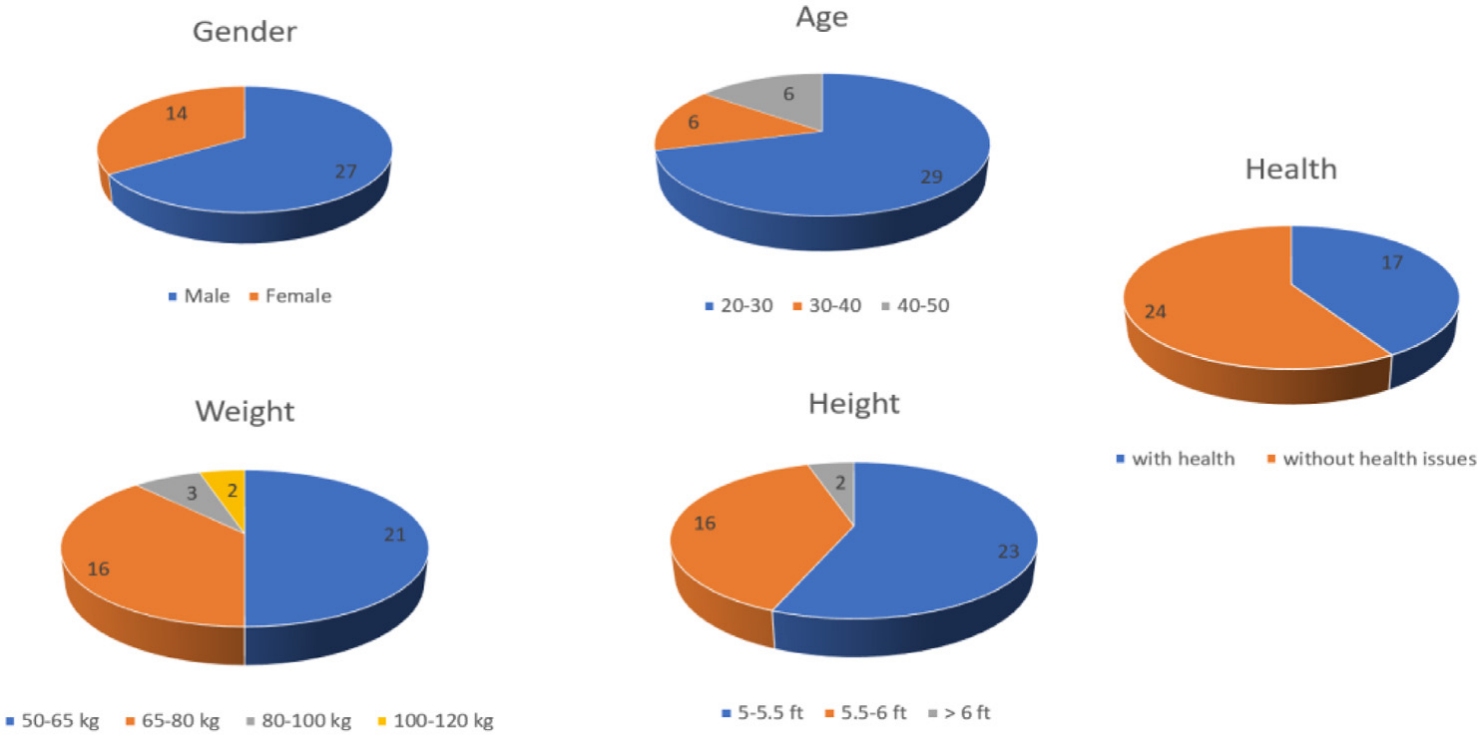
Volunteer statistics.

Volunteer statistics:

The 41 volunteers were drawn from a wide demographics and it was ensured that they varied in terms of Age, Height, Weight, Gender and pre-existing health conditions. The volunteer statistics is represented using a series of pie chart below in Fig. 5.

The complete details of the volunteers is given in Table 2.

**Table 2:** Volunteer details.

Subject Id	Gender	Height (cm)	Weight (KG)	Age	Heart Rate (base)	Health condition
1	Male	167.64	65	25	114	Sinus Tachycardia
2	Male	193.04	98	41	82	High Blood Pressure, Overweight
3	Female	152.4	62.50	46	79	No existing Health Issues
4	Female	157.48	50	23	110	Multiple allergies
5	Female	170.18	62	20	97	No existing Health Issues
6	Male	165.10	100	24	84	Obese
7	Male	162.56	62	24	65	No existing Health Issues
8	Male	172.72	74.50	24	78	No existing Health Issues
9	Male	165.10	80	26	70	overweight
10	Female	157.48	68	38	87	No existing Health Issues
11	Female	165.10	81	37	98	Thyroid, overweight
12	Male	170.18	63.50	21	60	No existing Health Issues
13	Male	170.18	65	25	85	No existing Health Issues
14	Male	154.94	80	21	100	Obese
15	Female	157.48	80	25	105	Obese
16	Female	157.48	55	24	110	No existing Health Issues
17	Female	162.56	74	25	103	No existing Health Issues
18	Female	162.56	70	23	86	No existing Health Issues
19	Female	157.48	79	21	104	Obese
20	Female	160.02	56	20	76	Hypochondria and extreme anxiety
21	Female	157.48	66	37	90	No existing Health Issues
22	Male	182.88	60	20	93	No existing Health Issues
23	Male	175.26	55	21	60	No existing Health Issues
24	Male	172.72	65.50	20	84	No existing Health Issues
25	Male	170.18	63.50	21	90	No existing Health Issues
26	Male	167.64	61	20	73	No existing Health Issues
27	Male	167.64	53	21	55	Low Blood pressure
28	Male	167.64	56	22	71	No existing Health Issues
29	Male	167.64	74	21	77	No existing Health Issues
30	Male	165.10	75	42	80	Arthritis
31	Male	162.56	50	44	80	No existing Health Issues
32	Female	157.48	61	20	85	No existing Health Issues
33	Female	157.48	50	22	109	Sinusitis
34	Male	180.34	68	38	93	Genetic Diabetes
35	Male	162.56	60	25	75	No existing Health Issues
36	Male	167.64	78	26	82	No existing Health Issues
37	Male	180.34	78	47	90	Diabetes and High Pressure
38	Male	165.10	71	41	75	High Blood pressure
39	Male	152.40	60	37	70	No existing Health Issues
40	Male	157.48	62	37	62	No existing Health Issues
41	Male	182.88	120	29	95	High blood pressure, Obese

## Limitations

Not applicable.

## Ethics Statement

As our work involved human subjects, an informed consent form as per our university guidelines was taken from every volunteer and witnessed by two people in a standard format provided by the university guidelines. All Female volunteer were supervised by a female faculty as per university guideline. Ethical approval of surveys were not required, only the institute's rules and regulation needed to be followed which we have.

## CRediT authorship contribution statement

**Purab Nandi:** Methodology, Formal analysis, Validation, Writing – original draft, Data curation. **K. R Anupama:** Conceptualization, Writing – original draft, Supervision, Project administration. **Himanish Agarwal:** Software, Validation, Visualization. **Kishan Patel:** Software, Formal analysis, Resources. **Vedant Bang:** Resources, Conceptualization. **Manan Bharat:** Resources, Data curation. **Madhen Vyas Guru:** Methodology, Visualization.

## Data Availability

BITS-2 Dataset for Fall Detection (Original data) (zenodo).
